# Osteopontin as a diagnostic and NTZ-response biomarker of multiple sclerosis: a systematic review and meta-analysis

**DOI:** 10.3389/fimmu.2025.1597117

**Published:** 2025-06-16

**Authors:** Adela González-Jiménez, Elena Urcelay, Laura Espino-Paisán

**Affiliations:** ^1^ Lab. Genetics and Molecular Bases of Complex Diseases, Health Research Institute of Hospital Clinico San Carlos (IdISSC), Madrid, Spain; ^2^ Cooperative Research Networks Oriented to Health Results (RICORS, REI), Health Research Institute Carlos III (ISCIII), Madrid, Spain

**Keywords:** osteopontin, multiple sclerosis, diagnostic biomarker, natalizumab, treatment response

## Abstract

**Introduction:**

Multiple sclerosis (MS) is a neuroinflammatory complex disease of the central nervous system (CNS). Diagnosing MS remains challenging due to its nonspecific signs, highlighting the need for reliable biomarkers. One potential biomarker is osteopontin (OPN), found in cerebrospinal fluid (CSF) and peripheral blood. This article presents a systematic review and meta-analysis of the association between OPN levels in CSF and blood and the presence of MS.

**Methods:**

We searched PubMed, Embase, and Cochrane databases for articles measuring OPN concentrations in peripheral blood and CSF samples from MS patients, published before July 12, 2024. A total of 605 articles were identified, and 29 were included in the analysis. Risk of bias was assessed with the NOS scale. The study protocol was officially registered in the PROSPERO website (registration number: CRD42023473406). We extracted standardized mean differences, 95% confidence intervals, and two-sided p values from each study and conducted a meta-analysis using a random-effects model. The heterogeneity among studies was evaluated by I-squared (I2), with values greater than 40% indicating high heterogeneity.

**Results and discussion:**

The present analysis revealed that individuals who suffered a first episode suggestive of MS, Clinically Isolated Syndrome (CIS), exhibited higher OPN levels in CSF than controls and patients with other neurological disorders (OND), emerging as an additional diagnosis tool. Furthermore, the observed decrease of OPN levels after Natalizumab (NTZ) treatment evidenced its potential as a biomarker of its efficacy. Higher OPN levels were found in CSF of individuals with MS compared to healthy controls (HC) and subjects with no other neurological diseases (NOND), result corroborated in relapsing remitting (RRMS) and secondary progressive (SPMS) patients. Similar OPN levels were observed when comparing MS patients to OND patients, suggesting that elevated OPN levels may be a common feature across various neurological conditions.

**Systematic Review Registration:**

https://www.crd.york.ac.uk/prospero/, identifier CRD42023473406.

## Introduction

Multiple sclerosis (MS) is a neurological autoimmune disease characterized by axonal demyelination leading to neurodegeneration. It affects over 2.5 million people worldwide, representing the primary cause of neurological disability in young adults (1). MS usually debuts as a clinically isolated syndrome (CIS) and shows varied clinical courses (2, 3), traditionally classified as relapsing-remitting MS (RRMS), secondary progressive MS (SPMS, as an evolution of the RRMS), and primary progressive MS (PPMS, only 10-15% of overall cases).

Early diagnosis is crucial in managing MS, yet remains challenging due to lack of specific biomarkers (4). Diagnosis typically involves magnetic resonance imaging (MRI) to detect and measure brain lesions, along with analysis of oligoclonal IgG bands in sera and cerebrospinal fluid (CSF) and evoked potentials (5). However, these techniques are invasive and costly, underscoring the need for new biomarkers in MS. MS misdiagnosis is common and a recent report found that near 20% patients with an established diagnosis of MS did not fulfill contemporary McDonald Criteria and had a more likely alternate diagnosis (6).

Osteopontin (OPN) emerges as a potential biomarker candidate. This multifunctional cytokine and adhesion molecule is involved in bone remodeling, wound healing, and immune cell activation (7, 8). OPN also plays a role in promoting a proinflammatory cytokine balance while inhibiting anti-inflammatory responses (9). OPN, produced by immune cells as well as neurons and glia, is therefore relevant in neuroinflammatory diseases like MS (10). Studies indicate that OPN levels are elevated in MS plaques dissected from patients’ brains, suggesting its involvement in disease pathogenesis (11). Additionally, OPN can suppress autoreactive T cells, potentially influencing immune-mediated diseases (12).

In the past two decades, over 25 therapies both injectable and oral were approved for RRMS, some with indications for progressive disease. However, patients with MS still experience progression of disability via neurodegeneration (13). Two alternative strategies have been proposed for MS treatment, namely escalation and early, or in some cases first-line treatment with high-efficacy agents. Escalation involves initiation of treatment with a drug of low or moderate efficacy, evaluation of treatment response and, if needed, switching to other medication. Safety concerns supported this approach, but the development of therapies with better safety profiles and the implementation of risk mitigation actions to limit side effects foster the use of highly effective therapies for early treatment (14). Patients initiating early treatment, regardless of prognostic factors and MRI burden at baseline, show significantly reduced disability progression (15). In fact, Natalizumab (NTZ), a therapy used in several diseases such as MS and Crohn’s disease (16), is a humanized monoclonal antibody targeted to the α4 integrin that diminishes T-cell trafficking to the CNS and reduces relapse rate. However, the effect of NTZ on OPN levels is not completely elucidated yet.

In summary, both early diagnosis and therapeutic intervention delay long-term disease progression, and the identification of diagnostic and response biomarkers is an unmet need. Given these observations, OPN seems to hold promise as a valuable biomarker for MS. Our aim is to conclusively evaluate the utility of OPN as a diagnostic biomarker in MS through systematic review of the literature and meta-analysis, and to assess the OPN potential aiding in NTZ treatment evaluation.

## Methods

### Search strategy, selection of studies, and data extraction

The present study followed the Preferred Reporting Items for Systematic Reviews and Meta-Analyses (PRISMA, **Supplementary Table 1**) (17, 18). The study protocol was officially registered with number CRD42023473406 on the PROSPERO website (https://www.crd.york.ac.uk/prospero/).

Review team members, AGJ and LEP, conducted independent searches in PubMed, EMBASE, and the Cochrane Library, according to the Peer Review of Electronic Search Strategies (PRESS) checklist (19), using the following Mesh terms: 1. Regarding population target: “Sclerosis, Multiple” OR (Sclerosis AND (Disseminated OR Acute)) OR (Sclerosis AND (Progressive OR Relapsing OR Remittent OR Secondary OR Primary)). 2. Regarding the possible biomarker: (“Osteopontin” OR “Sialoprotein 1” OR “Secreted Phosphoprotein 1” OR “Bone Sialoprotein 1” OR (Sialoprotein AND Bone) OR “Uropontin”). 1 and 2 were joined with the Boolean operator “AND”. Filters: Human, English, Spanish. With search strategies as depicted in **Supplementary Figure 1** and addressing the PICO question (www.training.cochrane.org/handbook.). All papers up to July 12, 2024 were compiled in an EndNote file to facilitate the identification and elimination of duplicates.

Original articles considered for inclusion, either report the baseline phase of clinical trials wherein OPN levels were measured in MS patients (including CIS, RRMS, SPMS, PPMS, or unspecified) or were observational studies. Articles in English or Spanish were included, and conference abstracts were also eligible for inclusion. Furthermore, each selected study included a control group, which consisted of healthy controls (HC), patients without neurological disorders (No Other Neurological Disorder, NOND), or patients with a neurological disorder different from MS (Other Neurological Disorder, OND).

Data from each selected study were meticulously extracted and recorded in an Excel file. To ensure accuracy, extracted data were cross-verified, and any inconsistencies were discussed and resolved between both review team members. The collected data included: author’s name, title, country of origin, year of publication, type of sample used for analysis (plasma, serum, or cerebrospinal fluid), method for OPN determination, number of controls and patients, age distribution, percentage of women in each group, MS clinical form, treatment details (if relevant), and OPN levels presented as mean with standard deviation (**Tables 1**, **2**, **3**). Trying to maintain homogeneity, only OPN determinations by enzyme-linked immunosorbent assays (ELISAs) were analyzed. When data were not provided in the original studies, corresponding authors were contacted via email to request the missing information.

**Table 1:** Review of the osteopontin (OPN) levels in multiple sclerosis (MS) and other neurological diseases (OND) patients (ELISA kit manufacturers provided in footnote).

Study and Sample population	Sample	Type	N	Age	% Women	OPN levels ng/mL (mean± SD)
2008; M. Braitch; UK (20)	Plasma^1^	MS	27	42.1 ± 10.1	62.96	380 ± 236
		OND	11	50.5 ± 15.8	37	386 ± 271
	CSF^1^	MS	27	42.1 ± 10.1	62.96	415 ± 186
		OND	11	50.5 ± 15.8	37	563 ± 411
2008; S.A Chowdury; USA (21)	CSF^1^	MS	30	24–71 years	60	7400 ± 6400
		OND	36	20–71 years	61	5800 ± 4400
2011; L. Bornsen; Sweden and Denmark (22)	Plasma^2^	CIS	24	Not shown	68	47.9 ± 15.69
		RRMS	38		56.09	43 ± 12.15
		SPMS	26		53.57	56 ± 11.71
		PPMS	9		55.55	64.35 ± 21.73
		OND	44	Not shown	59.99	51 ± 17.80
	CSF^2^	CIS	24	Not shown	68	150.33 ± 99.26
		RRMS	38		56.09	216 ± 132.59
		SPMS	26		53.57	163.33 ± 77.04
		PPMS	9		55.55	221.67 ± 38.52
		OND	44	Not shown	59.99	119 ± 66.76
2013; L.J. Edwards; UK and Germany (23)	CSF^2^	MS	40	42.5 (18-70)*	73.80	365.33x10^-3^ ± 49.20x10^-3^
		OND	8	67 (38-72)*	37.5	338.60x10^-3^ ± 48.6x10^-3^
2013; M. Khademi; Sweden (24)	CSF^2^	CIS	169	35.9 (16-65)*	74	116.9 ± 36.37
		RRMS	389	34.3 (17-73)*	71	140.4 ± 47.6
		SPMS	54	54.6 (35-81)*	61	134.9 ± 31.58
		PPMS	28	51.7 (35-67)*	50	127.6 ± 35.41
		OND	223	49.6 (13-83)*	74	144.6 ± 54.14
2013; Y. Shimizu; Japan (25)	Plasma^3^	MS relapse	17	38.3 ± 10.4	65	69.24 ± 36.85
		MS remission	17	38.3 ± 10.4	65	61.64 ± 16.31
		RRMS	11	Not shown	Not shown	53.39 ± 4.54
		SPMS	6	Not shown	Not shown	93.28 ± 19.76
		OND relapse	16	43.3 ± 10.3	96	76.86 ± 30.18
		OND remission	16	43.3 ± 10.3	96	52.72 ± 23.16
2015; Y. Kariya; Japan (26)	CSF^2^	MS	19	34.4 ± 10.8	94.73	156.5 ± 31.32
		OND	14	47.7 ± 19.1	86	133.2 ± 31.76
2017; C. Tortorella; Italy (27)	CSF^2^	CIS	41	32 ± 8	56.09	141.2 ± 93.8
		OND	30	37.7 ± 12.6	36.66	87 ± 74.2
2024; E. Kodosaki; UK (28)	Serum^4^	MS	77	41.9 ± 12.4	60	7.80x10^8^ ± 1.31x10^9^
		OND	80	33.2 ± 10.2	90	9.72x10^8^ ± 7.89x10^8^
	CSF^4^	MS	77	41.9 ± 12.4	60	1.39x10^8^ ± 1.98x10^9^
		OND	80	33.2 ± 10.2	90	1.02x10^8^ ± 9.05x10^9^

^1^TiterZymeR Enzyme Immunometric Assay, Assay Designs; ^2^R&D Systems Europe, Ltd., Abingdon, UK; ^3^R&D Systems, Minneapolis, USA; ^4^In house. *Median (range).

**Table 2:** Review of the osteopontin (OPN) levels in multiple sclerosis (MS), no other neurological diseases (NOND) patients, and healthy controls (ELISA it manufacturers provided in footnote).

Study and Sample population	Sample	Type	N	Age	% Women	OPN levels ng/mL (mean ± SD)
2005; A. Chioccheti; Italy (29)	Serum^1^	MS	71	Not shown	Not shown	221 ± 104.9
		HC	81	Not shown	Not shown	138.23 ± 73.53
2005; M.Comabella; Spain (30)	Plasma^2^	RRMS remission	46	35.6 ± 8.9	63	61.89 ± 24.70
		RRMS relapse	26	30.7 ± 9.9	69.2	80.87 ± 27.61
		SPMS	35	47.5 ± 9.6	80	86,39 ± 25,48
		PPMS	71	49.2 ± 8.3	50.7	63.34 ± 19.32
		HC	36	40.7 ± 11.2	52.8	66.06 ± 23.25
2008; M. Braitch; UK (20)	Plasma^2^	MS	27	42.1 ± 10.1	62.96	380 ± 236
		NOND	24	42.1 ± 18.7	75	377 ± 121
	CSF^2^	MS	27	42.1 ± 10.1	62.96	415 ± 186
		NOND	24	42.1 ± 18.7	75	286 ± 150
2009; A. Altintas; Turkey (31)	Plasma^2^	MS	50	37.4 ± 10.8	66	15.9 ± 36.2
		RRMS	33			11.3 ± 32.1
		SPMS	12			21.7 ± 42.7
		PPMS	5			30.4 ± 45.6
		HC	30	36.5 ± 8.8	80	155.4 ± 81.8
2011; M. Assadi; Iran (32)	Serum^3^	RRMS	35	31.57 ± 7.26	77.14	41.2 ± 2.35
		HC	38	30.06 ± 6.12	78.94	37.67 ± 2.46
2011; L. Bornsen; Sweden and Denmark (22)	Plasma^4^	CIS	24	Not shown	68	47.9 ± 15.69
		RRMS	38	Not shown	56.09	43 ± 12.15
		SPMS	26	Not shown	53.57	56 ± 11.71
		PPMS	9	Not shown	55.55	64.35 ± 21.73
		HC	24	Not shown	62.55	44 ± 110.39
	CSF^4^	CIS	24	Not shown	68	150.33 ± 99.26
		RRMS	38	Not shown	56.09	216 ± 132.59
		SPMS	26	Not shown	53.57	163.33 ± 77.04
		PPMS	9	Not shown	55.55	221.67 ± 38.52
		HC	24	Not shown	62.55	Not shown
2012; S. R Wen; China (33)	Serum^4^	RRMS	51	36.49 ± 5.15	70.58	43.13 ± 25.71
		NOND	48	35.20 ± 5.15	56.25	41.17 ± 14.21
	CSF^4^	RRMS	51	36.49 ± 5.15	70.58	199.75 ± 92.78
		NOND	48	35.20 ± 5.15	56.25	41.17 ± 14.21
2013; J Romme Christensen; Sweden and Denmark (34)	CSF^5^	RRMS	36	34 (31-40)**	55.55	237.14 ± 130
		SPMS	40	51 (44-57)**	52.5	171.43 ± 107.14
		PPMS	21	48 (38-53)**	52.38	192.86 ± 30
		NOND	20	53 (39-65)**	45	114.29 ± 54.29
2013; L. J. Edwards; UK and Germany (23)	CSF^4^	MS	40	42.5 (18-70)***	73.80	365.33x10^-3^ ± 49.20x10^-3^
		NOND	22	Not shown	95	235.08x10^-3^ ± 57.18x10^-3^
2013; L Szalardy; Hungary (35)	CSF^5^	MS	50/74	35.2 (18.3)**	62	140.6 ± 139.56
		CIS	23	Not shown	Not shown	123.9 ± 106.96
		RRMS	17	Not shown	Not shown	147.1 ± 184.07
		PPMS	10	Not shown	Not shown	202.3 ± 168
		NOND	19/30	36.3 (19.4)**	50	77.9 ± 50.96
2013; M. Khademi; Sweden (24)	CSF^4^	CIS	169	35.9 (16-65)***	74	116.9 ± 36.37
		RRMS	389	34.3 (17-73)***	71	140.4 ± 47.6
		SPMS	54	54.6 (35-81)***	61	134.9 ± 31.58
		PPMS	28	51.7 (35-67)***	50	127.6 ± 35.41
		NOND	203	41.1 (19-82)***	72	111.8 ± 40.14
2013; Y. Shimizu; Japan (25)	Plasma^5^	MS relapse	17	38.3 ± 10.4	65	69.24 ± 36.85
		MS remission	17	38.3 ± 10.4	65	61.64 ± 16.31
		RRMS	11	Not shown	Not shown	53.39 ± 4.54
		SPMS	6	Not shown	Not shown	93.28 ± 19.76
		HC	20	26.3 ± 2.8	70	33.76 ± 26.56
2014; P. Iaffaldano; Italy (36)	Plasma^4^	MS NTZ	49	34.23 ± 10.12	75.51	65.42 ± 22.20
		MS without treatment	24	35.8 ± 10.83	70.83	67.70 ± 24.23
		HC	22	39.18 ± 10.12	54.54	53.20 ± 12.68
2014; P. Kivisäkk; USA (37)	Plasma^6^	MS	492	45.9 ± 11.0	75	43.4 ± 0.9
		CIS	26	42.3 ± 9.6	85	Not shown
		RRMS	388	44.3 ± 10.3	78	43.6 ± 1.0
		SPMS	54	54 ± 10.5	68.5	45.2 ± 1.0
		PPMS	24	57.8 ± 10	46	Not shown
		HC	54	42.4 ± 12.8	72	37.2 ± 1.5
2015; M. Stilund; Denmark (38)	Serum(Kit undisclosed)	CIS	27	37(16-71)*	74	26.23 ± 14,43
		RRMS	44	37 (23-62)*	84	19.54 ± 8.65
		PPMS	15	53 (35-64)*	47	30.72 ± 18.6
		HC	39	41 (25-57)*	90	18.05 ± 7.40
	CSF(Kit undisclosed)	CIS	27	37(16-71)*	74	134.41 ± 257.18
		RRMS	44	37 (23-62)*	84	131.98 ± 292.68
		PPMS	15	53 (35-64)*	47	116.92 ± 348
		HC	39	41 (25-57)*	90	110.32 ± 273.88
2016; V Ferret Serna; Portugal (39)	Plasma^4^	RRMS	12	43.4 ± 12	100	104.1 ± 40.6
		HC	9	Not shown	100	51.1 ± 18
2017; I Hakansson; Sweden (40)	CSF^5^	MS	41	31 (24–36)*	78	8.3x10^-5^ ± 5.41x10^-5^
		HC	18/22	32 (26–41)*	77	6.5x10^-5^ ± 4.8x10^-5^
2017; S. Allahdadian; Iran (41)	Plasma(Method undisclosed)	RRMS	36	Not shown	Not shown	4.24 ± 1.209
		HC	35	Not shown	Not shown	3.41 ± 1.56
2018; P. Iaffaldano; Italy (42)	Plasma^4^	MS without treatment	10	36.8 ± 10.6	80	65.7 ± 24.3
		MS NTZ	10	30.8 ± 6.3	80	65.9 ± 16.6
		HC	10	42.7 ± 6.7	70	48.5 ± 7.8
2018; M. C. Gjelstrup; Denmark (43)	Serum^5^	CIS	10	34 (19-49)*	80	27.3 ± 4.75
		RRMS	25	36 (19-53)*	84	22.9 ± 3.85
		PPMS	5	64 (50-66)*	60	29.9 ± 15.8
		HC	20	41 (19-65)*	85	Not Shown
	CSF^5^	CIS	10	34 (19-49)*	80	64.1 ± 54.65
		RRMS	25	36 (19-53)*	84	81.4 ± 115.67
		PPMS	5	64 (50-66)*	60	98.6 ± 59.42
		HC	20	41 (19-65)*	85	Not Shown
2019; C. De Fino; Italy (44)	Serum^5^	CIS	25	37.4 (12.2)	28	9.1 ± 7.8
		RRMS	46	34.0 (9.2)	32.6	9.7 ± 6.9
		HC	11	38.8 ± 10.7	27.3	7.7 ± 5.8
	CSF^5^	CIS	25	37.4 (12.2)	28	32.2 ± 34.8
		RRMS	46	34.0 (9.2)	32.6	25 ± 19.9
		HC	11	38.8 ± 10.7	27.3	12.5 ± 7.4
2020; M. Jafarinia; Iran (45)	Plasma^7^	RRMS	40	32.30 ± 7.75	52.5	41.71 ± 11.61
		HC	38	31.82 ± 5.59	47.36	32.49 ± 17.77
2022; M. Golabi; Iran (46)	Plasma^8^	RRMS	30	38.36 ± 9.29	73.3	0.029 ± 0.042
		HC	45	38.4 ± 8.91	73.3	0.16 ± 0.022
2022; S. Kalinin; USA (47)	Serum^5^	RRMS	20	43.4 ± 7.58	100	8.45 ± 2.71
		HC	11	44.4 ± 2.9	100	8.9 ± 1.1

^1^Calbiochem, San Diego, CA; ^2^TiterZymeR Enzyme Immunometric Assay, Assay Designs; ^3^Biomedica Gruppe, Vienna, Austria; ^4^R&D Systems Europe, Ltd., Abingdon, UK; ^5^R&D Systems, Minneapolis, USA; ^6^Enzo Life Sciences, Farmingdale, NY; ^7^Glory Science Co., Ltd, USA; ^8^Abcam, USA. *Mean (range); **Median (IQR); ***Median (range).

**Table 3:** Review of the osteopontin (OPN) levels in multiple sclerosis (MS) before and after natalizumab treatment (NTZ).

Study and sample population	Sample	Type	N	Age	% Women	Treatment duration (months)	Baseline OPN levels ng/mL (mean ± SD)	OPN levels after NTZ treatment ng/mL (mean ± SD)
2014; J. Romme Christensen; Sweden and Denmark (48)	CSF^1^	SPMS	12	44(36–53)*	58.33	15	322 ± 33.16	65 ± 15.82
		PPMS	12	48 (27–55)*	50	15		
2014; P. Iaffaldano; Italy (36)	Plasma^2^	RRMS	49	34.23 ± 10.12	75.51	12	65.42 ± 22.20	55.23 ± 19.88
						18		32.81 ± 22.82
						24		45.96 ± 17.01
		MS without treatment	24	35.8 ± 10.83	70.83	Not shown	67.70 ± 24.23	Not shown
		HC	22	39.18 ± 10.12	54.54	Not shown	53.20 ± 12.68	Not shown
2016; V. Ferret Serna; Portugal (39)	Plasma^2^	RRMS	12	43.4 ± 12	100	3	104.1 ± 40.6	81 ± 24.5
						6		84.2 ± 29.1
		HC	9	Not shown	100	Not shown	51.1 ± 18	
2018; P. Iaffaldano; Italy (42)	Plasma^2^	RRMS	10	30.8 ± 6.3	80	12	65.9 ± 16.6	49.3 ± 20.0
		MS without treatment	10	36.8 ± 10.6	80	Not shown	65.7 ± 24.3	Not shown
		HC	10	42.7 ± 6.7	70	Not shown	48.5 ± 7.8	Not shown

Studies with a control group were also considered for the general analysis (ELISA kit manufacturers provided in footnote).

^1^R&D Systems, Minneapolis, USA; ^2^R&D Systems Europe, Ltd., Abingdon, UK; *Mean (range).

### Assessment of the quality of the selected studies

The review team members assessed the risk of bias in the selected studies using the Newcastle–Ottawa Scale (NOS) designed for nonrandomized studies. This scale evaluates the bias risk related to the selection and comparability of cases and controls, as well as the ascertainment of exposure, assigning a score within the range of 0 to 9. The outcomes of this assessment are presented in **Supplementary Table 2**, including the final NOS score obtained. Studies achieving a score between 7 and 9 were categorized as high-quality studies, while those with a score of 4–6 were classified as medium quality, and those with a score lower than 4 were considered low quality.

### Statistical analysis and strategy for data synthesis

All meta-analyses were conducted using Review Manager 5.4 (The Cochrane collaboration, 2020). The comprehensive synthesis of the included studies is presented in tables summarizing the key characteristics of each study (**Tables 4**–**6**). Standardized mean differences (SMD), 95% confidence intervals, and two-sided p values were extracted from each study and subjected to meta-analysis utilizing a random-effects model, with the I-squared (I^2^) statistic to assess heterogeneity among studies. A value of I^2^ greater than 40% was deemed indicative of high heterogeneity. Possible causes were explored through the analysis of various features in each study, and, if necessary, a reanalysis was carried out on smaller subgroups.

**Table 4:** Summary of meta-analysis undertaken with studies that compare MS and controls.

Comparison		Sample	Articles (ref)	I^2^ (%)	SMD (95% CI)	P-value
Case Group (type, n)	Control Group (type, n)					
MS (97)	OND (55)	CSF	Edwards 2013; Braitch 2008; Chowdury 2008	62	0.12 (-0.48, 0.73)	0.69
MS (44)	OND (27)	Plasma	Braitch 2008; Shimizu 2013	0	-0.12 (-0.61, 0.37)	0.62
MS (118)	HC and NOND (61)	CSF	Hakansson 2017; Szalardy 2013; Braitch 2008	0	0.55 (0.23, 0.87)	0.0007
MS (645)	HC and NOND (160)	Plasma	Atlintas 2009; Iaffaldano 2014; Iaffaldano 2018; Braitch 2008; Kivisak 2014; Shimizu 2013	99	1.11 (-1.57, 3.79)	0.42
RRMS (430)	OND (267)	CSF	Khademi 2013; Bornsen 2011	94	0.40 (-0.59, 1.39)	0.43
RRMS (52)	OND (60)	Plasma	Bornsen 2011; Shimizu 2013	0	-0.62 (-1.00, -0.23)	0.002
RRMS (532)	HC and NOND (292)	CSF	De Fino 2019: J Romme Christensen 2013; Khademi 2013; Szalardy 2013; Stilund 2015	54	0.58 (0.27, 0.88)	0.0002
RRMS (614)	HC and NOND (291)	Plasma	Atlintas 2009; Bornsen 2011; Comabella 2005; Jafarinia 2020; Golabi 2022; Kivisakk 2014; Allahdian 2017; Shimizu 2013; Ferret Serna 2016	99	0.42 (-1.35, 2.19)	0.64
RRMS (161)	HC and NOND (109)	Serum	De Fino 2019; Wen 2012; Kalinin 2022; Stilund 2015	0	0.24 (-0.01, 0.50)	0.06
PPMS (37)	OND (267)	CSF	Khademi 2013; Bornsen 2011	86	0.64 (0.29, 0.99)	0.0004
PPMS (86)	HC and NOND (281)	CSF	Romme Christensen 2013; Khademi 2013; Szalardy 2013; Stilund 2015	82	0.79 (0.11, 1.47)	0.02
PPMS (80)	HC and NOND (60)	Plasma	Bornsen 2011; Comabella 2005	0	-0.06 (-0.41, 0.30)	0.75
SPMS (80)	OND (267)	CSF	Khademi 2013; Bornsen 2011	87	0.19 (-0.60, 0.98)	0.64
SPMS (32)	OND (60)	Plasma	Bornsen 2011; Shimizu 2013	0	0.36 (-0.07, 0.80)	0.10
SPMS (94)	HC and NOND (223)	CSF	Romme Christensen 2013; Khademi 2013;	0	0.60 (0.33, 0.87)	<0.0001
SPMS (133)	HC and NOND (164)	Plasma	Atlintas 2009; Bornsen 2011; Comabella 2005; Kivisaak 2014; Shimizu 2013	98	1.52 (-0.63, 3.68)	0.17
CIS (65)	OND (74)	CSF	Tortorella 2017; Bornsen 2011	0	0.51 (0.16, 0.86)	0.004
CIS (217)	HC and NOND (233)	CSF	De Fino 2019; Khademi 2013; Szalardy 2013	32	0.29 (-0.03, 0.62)	0.07
CIS (52)	HC and NOND (50)	Serum	De Fino 2019; Stilund 2015	36	0.53 (-0.01, 1.06)	0.05

**Table 5:** Summary of meta-analysis undertaken with studies that include MS of different clinical forms.

Comparison		Sample	Articles (ref)	I^2^ (%)	SMD (95% CI)	P-value
Case Group (type, n)	Control Group (type, n)					
RRMS (549)	PPMS (88)	CSF	Romme Christensen 2013; Khademi 2013; Bornsen 2011; Szalardy 2013; Gjesltrup 2018; Stilund 2015	0	0.15 (-0.09, 0.39)	0.22
RRMS (71)	PPMS (14)	Plasma	Atlintas 2009; Bornsen 2011	52	-1.05 (-1.94, -0.16)	0.02
RRMS (69)	PPMS (20)	Serum	Gjesltrup 2018; Stilund 2015	0	-0.94 (-1.46, -0.42)	0.0004
RRMS (466)	SPMS (122)	CSF	Romme Christensen 2013; Khademi 2013; Bornsen 2011	35	0.32 (0.04, 0.60)	0.03
RRMS (499)	SPMS (135)	Plasma	Atlintas 2009; Bornsen 2011; Comabella 2005; Kivisak 2014; Shimizu 2013	88	-1.09 (-1.83, -0.36)	0.004
PPMS (419)	SPMS (120)	CSF	Romme Christensen 2013; Khademi 2013; Bornsen 2011	26	0.25 (-0.07, 0.57)	0.12
PPMS (14)	SPMS (38)	Plasma	Atlintas 2009; Bornsen 2011;	0	0.42 (-0.20, 1.04)	0.18
CIS (253)	RRMS (513)	CSF	Khademi 2013; Bornsen 2011; Szalardy 2013; Gjelstrup 2018; Stinlund 2015	30	-0.36 (-0.59, -0.13)	0.003
CIS (37)	RRMS (69)	Serum	Gjelstrup 2018; Stinlund 2015	0	0.72 (0.31, 1.13)	0.0007
CIS (253)	PPMS (67)	CSF	khademi 2013; Bornsen 2011; Szalardy 2013; Gjelstrup 2018; Stinlund 2015	0	-0.35 (-0.63, -0.07)	0.01
CIS (50)	PPMS (33)	Plasma	Bornsen 2011; Kivisakk 2014	33	-1.29 (-1.90, -0.67)	<0.0001
CIS (20)	PPMS (10)	Serum	Gjelstrup 2018; Stinlund 2015	0	-0.41 (-1.18, 0.36)	0.29
CIS (193)	SPMS (80)	CSF	Khademi 2013; Bornsen 2011	20	-0.40 (-0.73, -0.08)	0.01
CIS (48)	SPMS (52)	Plasma	Bornsen 2011; Kivisakk 2014	13	-0.36 (-0.78, 0.07)	0.10

**Table 6:** Summary of meta-analysis undertaken with studies that compare the effect of NTZ on OPN levels.

Comparison		Sample	Articles (ref)	I^2^ (%)	SMD (95% CI)	P-value
Case Group (type, n)	Control Group (type, n)					
MS pre-NTZ treatment (71)	HC (41)	Plasma	Ferret-Serna 2016, Iaffaldano 2014; Iaffaldano 2018	40	1.01 (0.41-1.61)	0.001
MS pre-NTZ treatment (71)	MS post-NTZ treatment (71)	Plasma	Ferret-Serna 2016, Iaffaldano 2014; Iaffaldano 2018	0	0.54 (0.21, 0.88)	0.002
MS post-NTZ treatment (71)	HC (41)	Plasma	Ferret-Serna 2016, Iaffaldano 2014; Iaffaldano 2018	58	0.40 (-0.28, 1.08)	0.25

## Results

We initially identified 605 articles using our search strategy. After eliminating 87 duplicate entries, the first phase of selection involved screening articles based on their titles and abstracts. A total of 454 articles were excluded as they did not address our objectives, leaving 64 articles for a more detailed evaluation. Subsequently, they were selected for systematic review and meta-analysis. Among these, nine were discarded as they were not performed in humans and 26 lacked sufficient information for inclusion. Four out of the 29 selected articles allowed the evaluation of NTZ efficacy (Flow diagram as shown in **Supplementary Figure 1**).

**Figure 1:**
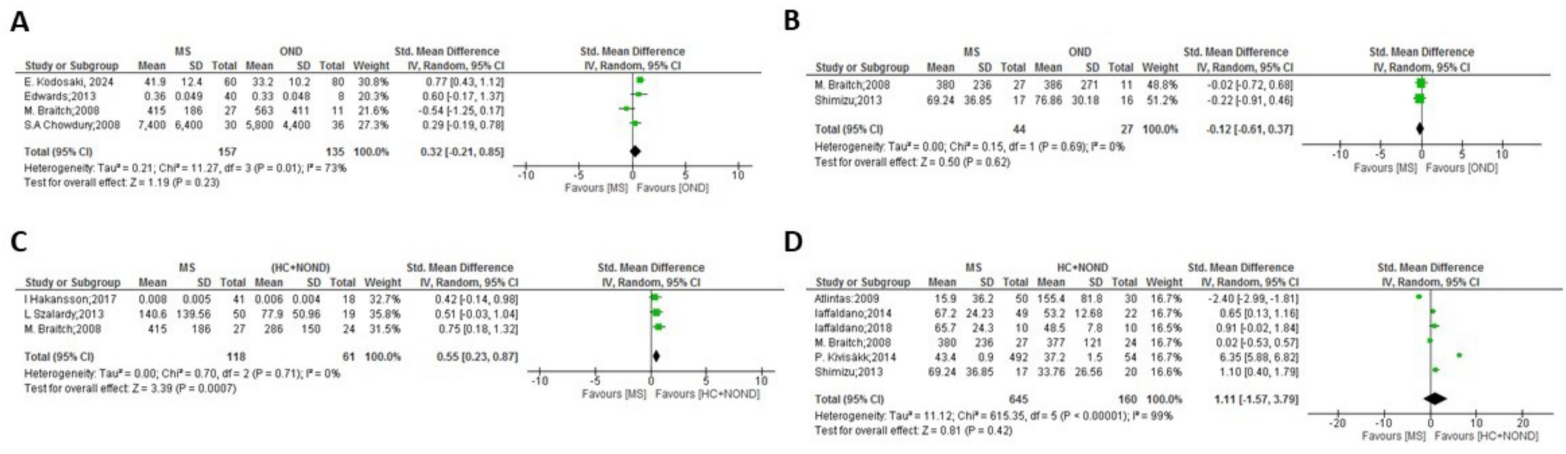
Meta-analysis of OPN levels in MS compared to controls: **(A)** MS patients versus OND in CSF. **(B)** MS patients versus OND in plasma. **(C)** MS patients versus HC and NOND in CSF. **(D)** MS patients versus HC and NOND in plasma.

**Figure 2:**
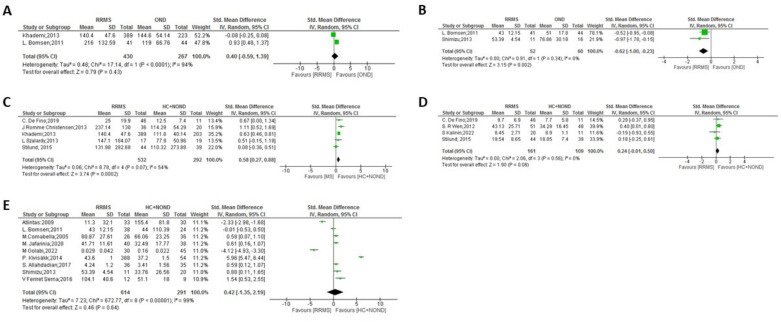
Meta-analysis of OPN levels in RRMS compared to controls: **(A)** RRMS patients versus OND in CSF. **(B)** RRMS patients versus OND in plasma. **(C)** RRMS patients versus HC and NOND in CSF. **(D)** RRMS patients versus HC and NOND in serum. **(E)** RRMS patients versus HC and NOND in plasma.

**Figure 3:**
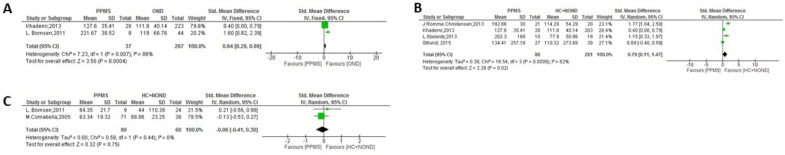
Meta-analysis of OPN levels in PPMS compared to controls: **(A)** PPMS patients versus OND in CSF. **(B)** PPMS patients versus HC and NOND in CSF. **(C)** PPMS patients versus HC and NOND in plasma.

**Figure 4:**
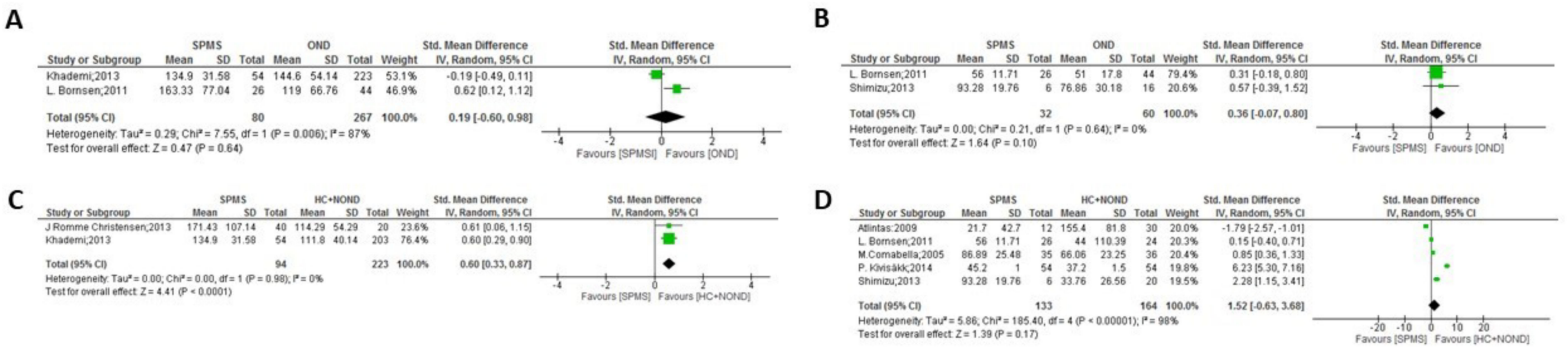
Meta-analysis of OPN levels in SPMS compared to controls: **(A)** SPMS patients versus OND in CSF. **(B)** SPMS patients versus OND in plasma. **(C)** SPMS patients versus HC and NOND in CSF. **(D)** SPMS patients versus HC and NOND in plasma.

**Figure 5:**
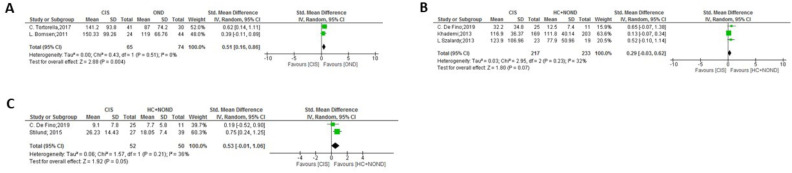
Meta-analysis of OPN levels in CIS compared to controls: **(A)** CIS patients versus OND in CSF. **(B)** CIS patients versus HC and NOND in CSF. **(C)** CIS patients versus HC and NOND in serum.

**Figure 6:**
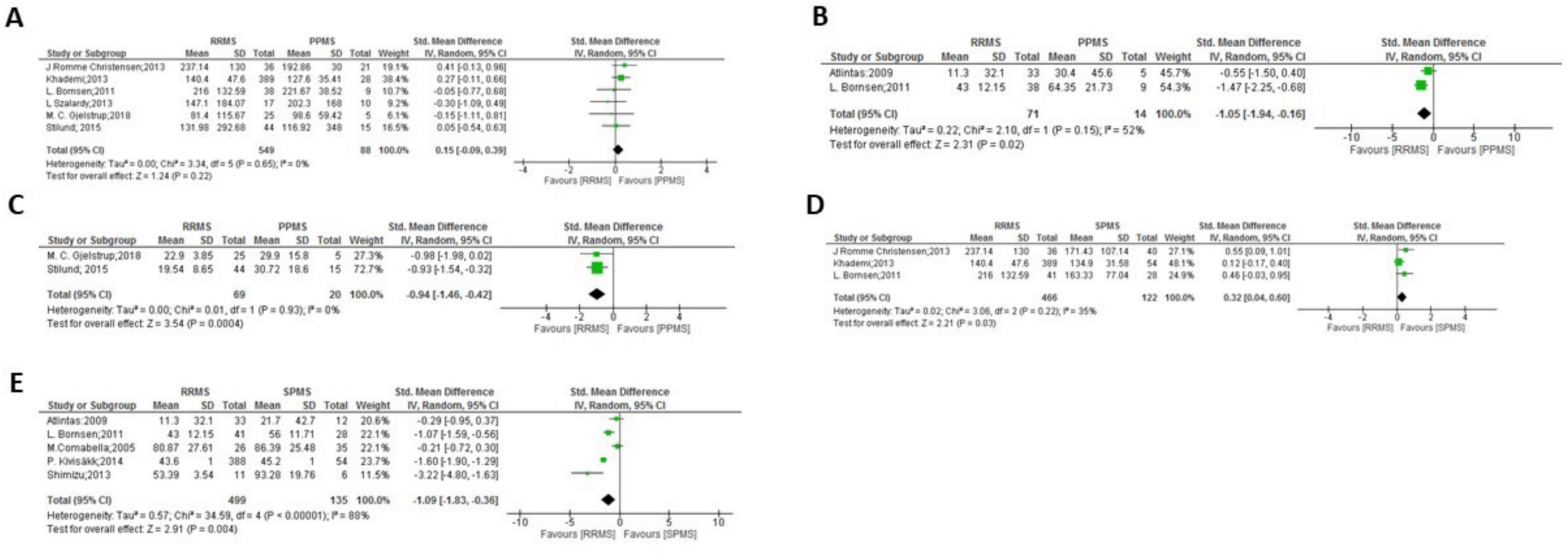
Meta-analysis of OPN levels in RRMS compared to progressive clinical forms: **(A)** RRMS patients versus PPMS patients in CSF. **(B)** RRMS patients versus PPMS patients in plasma. **(C)** RRMS patients versus PPMS patients in serum. **(D)** RRMS patients versus SPMS patients in CSF. **(E)** RRMS patients versus SPMS patients in plasma.

**Figure 7:**
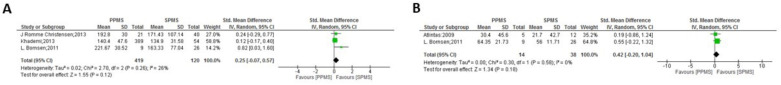
Meta-analysis of OPN levels between progressive clinical forms: **(A)** PPMS patients versus SPMS patients in CSF. **(B)** PPMS patients versus SPMS patients in plasma.

**Figure 8:**
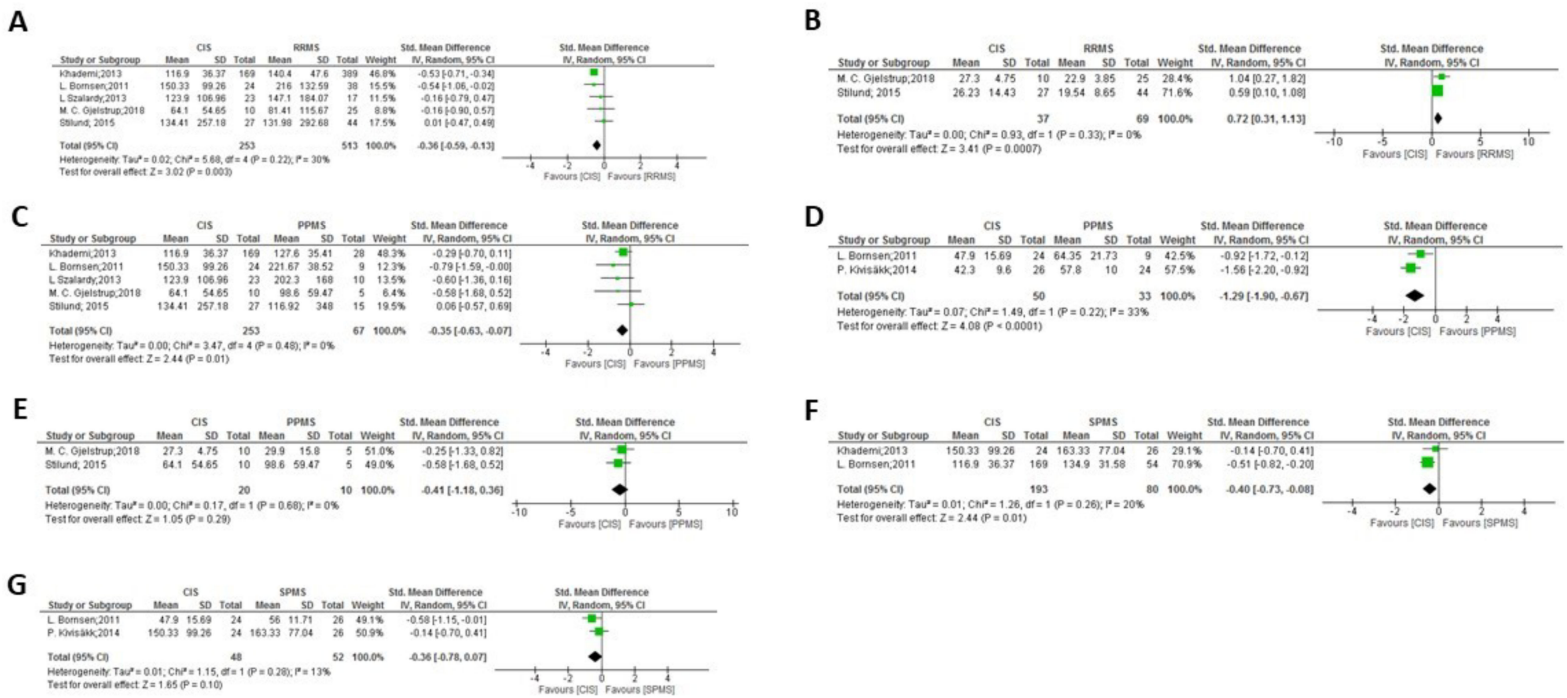
Meta-analysis of OPN levels in CIS compared to RRMS and progressive clinical forms: **(A)** CIS patients versus RRMS patients in CSF. **(B)** CIS patients versus RRMS patients in serum. **(C)** CIS patients versus PPMS patients in CSF. **(D)** CIS patients versus PPMS patients in plasma. **(E)** CIS patients versus PPMS patients in serum. **(F)** CIS patients versus SPMS patients in CSF. **(G)** CIS patients versus SPMS patients in plasma.

**Figure 9:**
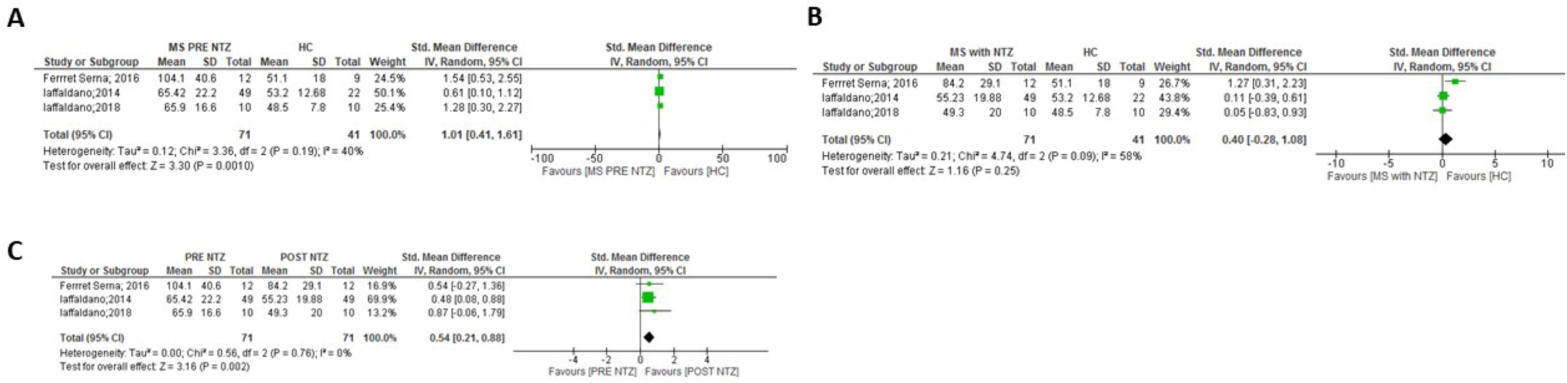
Meta-analysis of OPN levels in NTZ treatment. **(A)** MS before NTZ treatment versus HC in plasma. **(B)** MS patients treated with NTZ versus HC patients in plasma. **(C)** MS patients before and after NTZ treatment in plasma.

Data were extracted and compiled in **Tables 1**–**3**. Due to the similarity between HC and NOND groups, both were analyzed together. In the meta-analyses, levels of OPN in CSF, plasma, and serum samples were compared in MS patients with all types of controls. However, not all comparisons were feasible due to the limited availability of studies, particularly in serum samples. In addition, stratification by clinical forms and the effects of NTZ treatment on OPN levels were assessed. The results, including Chi-squared (χ²), I^2^, and Standard Mean Difference (SMD) were documented in **Figures 1**–**9**, as follows.

In the comparisons between overall MS and control groups, significant differences in OPN levels in CSF samples were observed only when comparing MS patients vs. HC and NOND (**Figure 1C**, p = 0.0007), with OPN levels being higher in MS patients. Similar trends were found when comparing each clinical form to HC and NOND, with significant differences found in CSF in most cases (p = 0.0002 for RRMS, p = 0.02 for PPMS, and p < 0.0001 for SPMS, **Figures 2C**, **3B**, **4C**, respectively). Even though the heterogeneity among studies was very high in the comparison of PPMS (**Figure 3B**, I^2^ = 82%), patients consistently showed increased OPN levels in all the studies included. The difference was also evident when comparing CIS with HC and NOND in serum samples (**Figure 5C**, p = 0.05), with a similar trend in CSF (**Figure 5B**, p= 0.07). However, these comparisons could not be evaluated in plasma samples, due to substantial heterogeneity among studies (**Figures 1D**, **2E**, **4D**). Regarding individuals with OND, the differences emerged in the comparison to each MS clinical form independently. Comparing plasma samples, RRMS patients displayed lower levels than controls (**Figure 2B**, p= 0.002), and similar trends were found when CSF samples of OND were compared to PPMS (**Figure 3A** ,p= 0.0004) and CIS (**Figure 5A**, p = 0.004). Significant differences were not detected when overall MS were considered (**Figures 1A, B** in CSF and plasma, respectively). Again, the high heterogeneity in CSF (I^2^ = 73%) hampered this evaluation, although three out the four studies included presented higher CSF levels in MS patients (**Figure 1A**).

In the comparison among clinical forms, significant differences were observed between RRMS and both PPMS (**Figures 6B, C**: p= 0.02 in plasma and p= 0.0004 in serum) and SPMS (**Figures 6D,E**: p= 0.03 in CSF and p= 0.0004 in plasma, with high heterogeneity but consistently displaying increased values in SPMS in the latter). No significant differences were found between the progressive forms (**Figure 7**). Lower levels of OPN were detected in CIS compared to MS clinical forms (**Figure 8**), with significant differences observed in the comparisons with RRMS (**Figure 8A**: p= 0.003 in CSF) and PPMS (**Figures 8C, D**: p= 0.01 in CSF and p < 0.0001 in plasma). An exception was noticed in the comparison between OPN levels in serum of CIS and RRMS patients, with higher levels in people with CIS compared to RRMS (**Figure 8B**: p= 0.0007).

Finally, a meta-analysis focused on Natalizumab (NTZ) treatment in MS patients was conducted (**Figure 9**), specifically in plasma samples. When comparing OPN levels before and after a minimum of 6 months of NTZ treatment, a significant reduction in OPN levels was observed with the presence of NTZ (**Figure 9C**: p = 0.002). After the 6 month-treatment, the comparison between OPN levels in RRMS patients and HC was not significant (**Figure 9B**), by decreasing the high OPN levels in pre-treatment patients and therefore canceling the significant difference initially observed with HC (**Figure 9A**, p= 0.001).

## Discussion

OPN is a pleiotropic protein able to regulate numerous pathways by interacting with multiple receptors. Osuka et al. (49) showed that thrombin cleavage of the full-length secreted OPN generates C- and N-terminal fragments, which exhibit distinct biological activities derived of the exposure of regions not accessible in the full-length molecule. The overexpression of OPN correlate with autoimmune disease severity (50). Nonetheless in MS, a multifactorial condition with elevated OPN levels, evidence also supports its neuroprotective effects (51, 52). The present study conducted a systematic review and meta-analysis of OPN levels as biomarker in MS. A prior revision in 2018 (53) reported that OPN could serve as a diagnostic and prognostic biomarker with clinical utility. However, the authors did not address the influence of treatments such as NTZ on OPN levels. Additionally, from 2018 several new studies on OPN levels were performed and, therefore, our study aimed to evaluate the potential use of OPN as diagnostic and treatment biomarker in MS. Our main purpose with this work was to restrict the analyses to an accessible and well-established OPN quantification method (ELISA), which could be easily incorporated to the clinical setting when validated as diagnostic marker. The reviewed studies employed CSF, plasma, and serum as sample sources. Most of them analyzed two types of samples to provide complementary results. While CSF collection is more invasive as compared to plasma and serum, sometimes it yields a more sensitive detection and therefore, studies typically employed CSF alongside alternations between serum and plasma. The studies exhibited significant variability and heterogeneity, posing a considerable challenge to extract firm conclusions. Upon closer examination of individual articles, disparities in the study design and sample processing surfaced, emphasizing these critical considerations for potential biomarker development.

Given the inherent diversity in clinical symptomatology and disease courses in MS, our meta-analyses used a random-effects model, which postulates that the actual impact of one variable may vary across studies owing to their heterogeneity (54). Certain comparisons excluded some studies. For instance, although MS is more prevalent in women (3:1), the study by Kariya et al. (26) was omitted as it presents higher percentage of women than the other studies included in the comparison of CSF between MS and other neurological disorders (OND) and, even then, the heterogeneity remained high (changed: from 92% to 62%). On the contrary, the study by De Fino et al. (44) was excluded due to a low percentage of women in the comparison between CIS and RRMS, decreasing heterogeneity from 62% to 30% in CSF and from 71% to 0% in serum. Furthermore, in the comparison of CSF between MS and the combination of HC and NOND, the study by Edwards et al. (23) was disregarded due to the use of a different ELISA kit and heterogeneity fell from 88% to 0%. Nonetheless, the cause of heterogeneity among articles could not be ascertained in some cases. For example, the study of Wen et al. (33) was disqualified in the comparison of CSF between RRMS and the combination of HC and NOND, resulting in a change in heterogeneity from 90% to 54%. Additionally, the article by Atlintas et al. (31) was excluded from the comparison of plasma between PPMS and HC and NOND, reducing heterogeneity from 75% to 0%. Finally, the study by Comabella et al. (30) in plasma was eliminated in the comparisons between RRMS and PPMS (heterogeneity changed from 92% to 52%) and PPMS vs. SPMS (heterogeneity changed from 87% to 0%). Significant difference in CSF levels of OPN exists between individuals with MS and the combined group of HC and NOND, showing higher levels in the MS group (**Figure 1C**); however, when comparing OPN levels between MS and patients with OND, this significant difference disappears (**Figure 1A**). Even after careful analysis, in this case the heterogeneity could not be resolved (**Figure 1A**, I^2^ = 73%) as the four studies presented analogous weight, all but the latter were performed in British population (the last one in North American population), and the two last ones used the same ELISA kit. Therefore, no clear reason exists to eliminate any of them. Although the mentioned heterogeneity hampers a conclusive interpretation and in the three studies with higher size out of the four studies included the OPN levels are increased in MS patients, the overall results would suggest that higher OPN levels might be a shared characteristic in various neurological disorders, irrespective of the specific condition. Notably, neurological disorders like Alzheimer’s and Parkinson’s diseases exhibit altered OPN levels (55–58). Consequently, as comparison of OPN levels between people with MS and OND rendered no differences, this biomarker would seem to lack enough specificity for a differential diagnosis of MS when other neurological conditions are considered, and OPN should be combined with additional tests to gain specificity for MS diagnosis. In this sense, Kodosaki et al. (28) claimed that combinations of biomarkers were considerably better than single biomarker predictions and specifically the combination of CSF [chitinase-3-like-1 + TNF-receptor-1 + CD27] and serum [osteopontin + MCP-1] presented an area under the curve of 0.97 for MS diagnosis. Interestingly, when stratifying by clinical form, significant differences become evident between OND and PPMS patients but not with RRMS or SPMS (**Figures 3A**, **2A**, **4A**, respectively), although these comparisons show the previously discussed heterogeneity among studies. Nonetheless, OPN levels would still act as a crucial diagnostic biomarker if able to distinguish the first episode of MS (CIS) from other neurodegenerative diseases. Our study suggests that high OPN levels could serve as potential biomarker for MS diagnosis, as they are higher in CIS CSF compared to OND individuals and to HC+NOND (**Figures 5A,C**), with two different studies included in each comparison. To advance in the validation of OPN as a potential biomarker for diagnosing MS at onset, next steps should emphasize the use of ELISA as a uniform methodology to ensure reliable comparisons and allow the definition of sensitivity and specificity for OPN detection.

Regarding the clinical forms, higher levels of OPN were detected in PPMS patients compared to RRMS in both serum and plasma samples (**Figures 6B, C**) and, even with high heterogeneity, SPMS showed higher plasma levels than RRMS (**Figure 6E**), suggesting that circulating OPN was elevated in both progressive MS forms, while RRMS showed higher OPN levels than SPMS in CSF (**Figure 6D**), as previously observed by Agah et al. (53). No significant differences were found between the progressive forms, PPMS and SPMS (**Figure 7**). No actual MS treatment stops neurodegeneration (59), which accumulates as the disease progresses, and circulating OPN seems indicative of this process. In a study not considered in the present meta-analysis as it did not include a control group and used a multiplex assay, Nowak-Kiczmer et al. reported significant differences in serum content of OPN between PPMS and either SPMS or RRMS, with the highest values present in SPMS and the lowest in PPMS (60). These differences underscore the importance of considering not only the specific clinical forms when exploring biomarkers for MS, but also the sample source and OPN determination method. In summary, the difference in OPN levels between MS clinical forms may aid in their accurate distinction, something important to ascertain underlying mechanistic processes (61, 62) and therefore, to suggest therapeutic strategies. When compared to the specific clinical forms, OPN levels in CSF of CIS individuals with a first episode were significantly lower than those found in RRMS, SPMS or PPMS patients (**Figures 8A, F, C**), replicating results previously observed by Agah and cols (53). In fact, factors including aging and OPN have been reported to cause microglia to lose their protective states and become injurious (63, 64).

Finally, our study aimed to evaluate the effect of NTZ on OPN levels. OPN synergistically interacts with Vascular Cell Adhesion protein 1 (VCAM-1) and facilitates lymphocyte homing to the inflamed brain in a process driven by the α4β1 integrin, which binds both VCAM-1 and OPN (10). The blockade of this integrin with the humanized monoclonal antibody, Natalizumab, reduces the relapse rate in MS. All studies included in the meta-analysis used plasma samples, except for the one with CSF samples conducted by Romme-Christensen et al. (34), which was consequently excluded from our meta-analysis. Before NTZ treatment, MS patients showed significantly higher OPN levels in plasma than HC (**Figure 9A**), and MS patients normalized OPN levels to those found in HC after NTZ treatment (between 6 and 12 months, **Figure 9B**). Notable differences emerged in MS patients following the administration of NTZ (**Figure 9C**), specifically after a minimum of 6 months of treatment, and our findings suggest that OPN could serve as a biomarker for evaluating the effectiveness of treatment, as expected provided the direct functional link of OPN with this MS therapy. In other MS therapeutic approaches, no sufficient number of studies associated OPN and treatment efficacy, although some isolated examples in certain therapies can be found (i.e (65, 66).

One of the caveats regarding the OPN evidence as a biomarker (as with many others), is the diversity of methodological approaches in the different studies. The present systematic review and meta-analysis considered the method of OPN determination, and only included studies that evaluated OPN levels through a widespread used method as ELISA. Despite this, up to seven different commercial and one in-house ELISA kits were counted in the reviewed studies, and a remarkable variability of reported levels was common among them. Before any possible implementation in the clinical setting, efforts should focus in the evaluation of the technique and laboratory tests that most accurately measure OPN levels in the diverse sample types, as discrepancies between OPN sample sources have been already described (61). To foster the use of OPN as a potential biomarker for MS, additional data are required. For instance, expanding the scope of the studies to include diverse ethnicities would enlarge the availability of study populations for comparison and facilitate the extrapolation of results beyond the Caucasian population.

Our work contributes with valuable insights to the ongoing efforts in identifying robust and clinically relevant biomarkers for MS, emphasizing the nuanced nature of the disease and the potential utility of OPN as additional discriminating factor for diagnosis. Further research to conclusively elucidate the specific roles and implications of OPN in the various clinical forms, will ultimately advance our understanding of MS pathophysiology and refine diagnostic approaches.

## Conclusions

- While this glycoprotein could be a potential biomarker for MS, levels of OPN show significant heterogeneity across comparisons and further efforts are required to standardize methodologies and to reduce the variability in sample sources.
- CIS cases exhibit significantly higher OPN levels in CSF than overall controls, pointing to an interesting additional diagnosis biomarker.
- Overall MS patients (and consistently the different clinical forms) present significantly higher levels of OPN in CSF than the combined group of healthy controls and no other neurological diseases, but only PPMS patients present higher levels in CSF than patients with other neurological diseases.
- In MS patients, NTZ treatment significantly reduces OPN plasma levels, which act as a biomarker for evaluating its effectiveness.
- These findings underscore the importance of considering specific clinical forms when exploring biomarkers for MS, and highlights the potential utility of OPN as a complementary tool in diagnosing and managing the disease.
- Additional studies are necessary to fully standardize OPN as MS biomarker, and to ultimately enhance diagnostic approaches in clinical practice.

## Data Availability

The original contributions presented in the study are included in the article/**Supplementary Material**. Further inquiries can be directed to the corresponding author.

## Supplementary material

Supplementary Figure 1 Flow diagram of the systematic review.

Supplementary Table 1 PRISMA checklist.

Supplementary Table 2 NOS score.
