# Supplementary figures and images for: Osteopontin as a diagnostic and NTZ-response biomarker of multiple sclerosis: a systematic review and meta-analysis
Source: Front Immunol. 2025 Jun 16;16:1597117. doi: 10.3389/fimmu.2025.1597117 (PMC12206654; doi:10.3389/fimmu.2025.1597117)

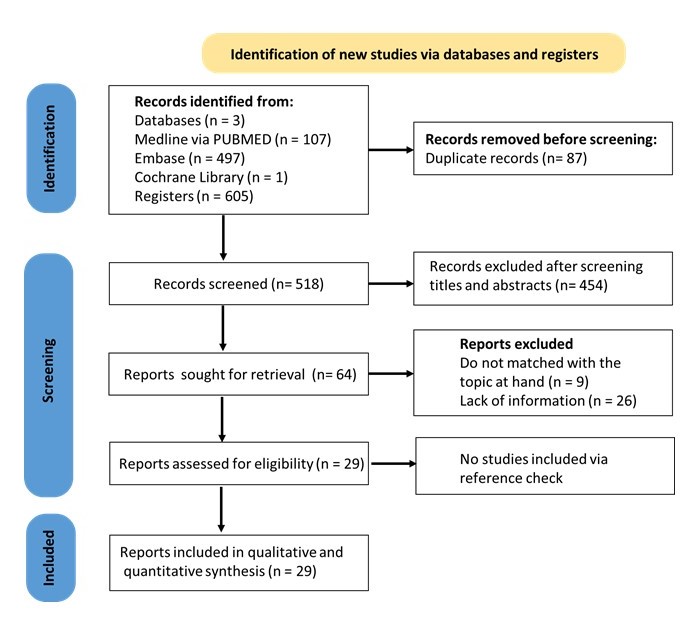

Supplement: Supplementary Figure 1 — Flow diagram of the systematic review. [file Image1.jpeg]
