# Supplementary material for: Osteopontin as a diagnostic and NTZ-response biomarker of multiple sclerosis: a systematic review and meta-analysis
Source: Front Immunol. 2025 Jun 16;16:1597117. doi: 10.3389/fimmu.2025.1597117 (PMC12206654; doi:10.3389/fimmu.2025.1597117)
Supplement: Supplementary Table 2 — NOS score. [file Table2.docx]

| **Article (Year; Author)** | **Selection (max. 4 stars)** | **Comparability (max. 2 stars)** | **Outcome (max. 3 stars)** | **Total**  **(max. 9 stars)** |
| --- | --- | --- | --- | --- |
| **2005; A. Chioccheti** | ★★★★ | ★ | ★★★ | 8 |
| **2005; M. Comabella** | ★★★★ | ★ | ★★★ | 8 |
| **2008; M. Braitch** | ★ | ★ | ★★★ | 5 |
| **2008; S. A. Chowdury** | ★★ | ★ | ★★★ | 6 |
| **2009; A. Atlintas** | ★★ | ★★ | ★★★ | 7 |
| **2011; M. Assadi** | ★★★★ | ★★ | ★★★ | 9 |
| **2011; L. Bornsen** | ★★ | ★ | ★★★ | 6 |
| **2012; S. R. Wen** | ★★ | ★★ | ★★★ | 7 |
| **2013; L. J. Edwards** | ★★ | ★ | ★★★ | 6 |
| **2013; J. Romme Christensen** | ★★★ | ★ | ★★★ | 7 |
| **2013; M. Khademi** | ★★★★ | ★★ | ★★★ | 9 |
| **2013; L. Szalardy** | ★★ | ★ | ★★★ | 6 |
| **2013; Y. Shimizu** | ★ | ★ | ★★★ | 5 |
| **2014; P. Iaffaldano** | ★★ | ★★ | ★★★ | 7 |
| **2014; P. Kivisäkk,** | ★★★ | ★ | ★★★ | 7 |
| **2015; M. Stilund** | ★★★ | ★ | ★★★ | 7 |
| **2015; Y. Kariya** | ★ | ★★ | ★★★ | 6 |
| **2016; V. Ferrret Serna** | ★★ | ★ | ★★★ | 6 |
| **2017; C. Tortorella** | ★★ | ★ | ★★★ | 6 |
| **2017; I. Hakansson** | ★★ | ★★ | ★★★ | 7 |
| **2017; S. Allahdadian** | ★★ | ★★ | ★★★ | 7 |
| **2018; P. Iaffaldano** | ★★ | ★ | ★★★ | 6 |
| **2018; M. C. Gjelstrup** | ★★ | ★★ | ★★★ | 7 |
| **2019; C. de Fino** | ★ | ★★ | ★★★ | 6 |
| **2020; M. Jafarinia** | ★★★ | ★★ | ★★★ | 8 |
| **2021; D. Marastoni** | ★ | ★ | ★★★ | 5 |
| **2022; M. Golabi** | ★★★★ | ★★ | ★★★ | 9 |
| **2022; S. Kalinin** | ★ | ★★ | ★★★ | 6 |
| **2024; E. Kodosaki** | ★★ | ★ | ★★★ | 6 |

**Supplementary Table 2: Quality assessment of the included studies** **using the Newcastle–Ottawa Scale (NOS)**
